# Low-tube-potential ultra-high-resolution coronary CTA with photon-counting detector CT for stent evaluation: a comparative feasibility study

**DOI:** 10.1007/s11604-025-01846-x

**Published:** 2025-08-09

**Authors:** Suguru Araki, Satoshi Nakamura, Shiko Okabe, Masafumi Takafuji, Yasutaka Ichikawa, Hajime Sakuma, Kakuya Kitagawa

**Affiliations:** 1 Department of Radiology, Mie University Hospital, 2-174 Edobashi, Tsu, Mie 514-8507 Japan; 2 Department of Advanced Diagnostic Imaging, Mie University Graduate School of Medicine, 2-174 Edobashi, Tsu, Mie 514-8507 Japan; 3 Regional Co-Creation Deployment Center, Mie Regional Plan Co-Creation Organization, Mie University, 1557 Kurimamachiyacho, Tsu, Mie 514-8507 Japan

**Keywords:** Coronary artery disease, Computed tomography, Stents, Radiation dosage

## Abstract

**Objectives:**

This study aimed to evaluate the feasibility of low-tube-potential ultra-high-resolution (UHR) coronary CT angiography (CCTA) using photon-counting detector CT (PCD-CT) in comparison to low-tube-potential CCTA using energy-integrating detector CT (EID-CT), with a specific focus on the evaluation of coronary stents.

**Materials and methods:**

This retrospective study included 54 patients (88 stents) who underwent CCTA in UHR mode on PCD-CT at low tube potential (PCD_UHR_-low; 27 patients [45 stents]) or CCTA with EID-CT at low tube potential (EID-low; 27 patients [43 stents]). The EID-low cohort was selected by propensity score matching to the PCD_UHR_-low cohort. Image quality of in-stent lumen was assessed using a 4-point Likert scale, with a score of 4 indicating “excellent.” Quantitative assessment of stents included stent-induced blooming, edge sharpness, stent full width at half maximum (FWHM) and ΔHU_in-stent_ which quantifies the increase in CT attenuation within the stent lumen. Radiation dose was evaluated using CT dose index volume (CTDIvol) and dose–length product (DLP).

**Results:**

PCD_UHR_-low had higher image quality scores than EID-low (scores of 3 and 4, 82.2% vs. 53.5%; *p* = 0.02). PCD_UHR_-low showed reduced stent-induced blooming, superior edge sharpness, smaller stent FWHM and smaller ΔHU_in-stent_ than EID-low (all, *p* < 0.01). CTDIvol and DLP in PCD_UHR_-low (9.3 ± 4.4 mGy and 107.1 ± 50.4 mGy・cm) were comparable to those of EID-low (10.5 ± 5.9 mGy and 118.2 ± 74.9 mGy・cm), respectively. (*p* = 0.26 and 0.47).

**Conclusion:**

CCTA using UHR mode in PCD-CT at low tube potential achieved superior image quality for implanted stent visualization as compared to EID-CT at low tube potential, while maintaining comparable radiation doses.

**Supplementary Information:**

The online version contains supplementary material available at 10.1007/s11604-025-01846-x.

## Introduction

Cardiac computed tomography (CT) is widely used as a noninvasive imaging modality in clinical practice [1, 2]. Coronary CT angiography (CCTA) can rule out coronary artery disease (CAD) with a high negative predictive value [3], and recent guidelines recommend CCTA as the modality of choice for evaluating coronary arteries in patients with stable chest pain and low to intermediate risk of coronary artery disease [4]. Despite its widespread utilization, the evaluation of coronary stenosis in the presence of coronary stents has been limited when using conventional energy-integrating detector CT (EID-CT) scanners. This limitation is mainly due to stent-induced blooming and beam-hardening artifacts, which compromise the diagnostic value of CCTA [5–8]. Stent-induced blooming has been primarily attributed to partial volume averaging, which is influenced by spatial resolution and detector cell size [9, 10], leading to inaccurate evaluation of stenosis. The structural limitations of EID-CT detector elements restrict spatial resolution and accurate delineation of stented segments, indicating the need for novel detector technologies.

Recently, photon-counting detector CT (PCD-CT) scanners equipped with dual-source CT technology have been introduced into clinical practice. A key advantage of PCD-CT is its improved photon efficacy, largely attributable to its spectral capabilities and the ability to exclude electronic noise. By applying appropriate energy thresholds, PCD-CT can effectively remove electronic noise from the signal, thereby improving the signal-to-noise ratio (SNR) and potentially reducing radiation dose [11, 12]. In addition, PCD-CT provides higher spatial resolution than EID-CT, particularly in its ultra-high-resolution (UHR) mode. The absence of septa between detector pixels in PCD-CT enables a slice thickness of 0.2 mm in UHR mode, reducing partial volume effects and improving stent-induced blooming artifacts [13–15]. Prior investigations have optimized UHR imaging parameters such as the choice of reconstruction kernels and the use of quantum iterative reconstruction (QIR) algorithms [16, 17].

However, while the previous studies have addressed several aspects of UHR imaging, the feasibility and utility of low-tube-potential protocols in UHR mode remain insufficiently explored. Tube potential affects contrast-to-noise ratio (CNR), the attenuation profiles of plaques and stents, and overall image quality. On the radiation dose side, the narrow X-ray beam width required by UHR mode [18] may lead to higher radiation exposure as compared to normal resolution mode. Considering these, it is desirable to achieve UHR-mode imaging at as low a dose as possible without compromising image quality.

Therefore, the aim of this study was to evaluate the image quality and radiation dose of low tube potential CCTA using PCD-CT UHR mode (PCD_UHR_-low) in the presence of coronary stents, and to compare these results with those obtained using EID-CT at low tube potential (EID-low), with the EID-low cohort selected by propensity score matching to the PCD_UHR_-low cohort. Through this comparative assessment, we seek to clarify the potential benefits of low tube potential and thereby guide more effective clinical use of UHR-mode PCD-CT for stent assessment.

## Materials and methods

### Study design

This retrospective, single-center study focused on the assessment of UHR-CCTA with a low tube potential of 70 or 90 kVp using a PCD-CT scanner. The institutional review board of our hospital approved the study protocol and waived the need for individual consent due to its retrospective design.

Between October 2023 and September 2024, 92 patients underwent CCTA for coronary artery stent evaluation using a PCD-CT scanner (NAEOTOM Alpha, Siemens Healthineers, Forchheim, Germany). Thirteen patients with a history of coronary artery bypass grafting (CABG) were excluded. Of the remaining 79 patients, 43 were further excluded because their scans were performed with normal resolution mode or with ECG-gated retrospective helical scans in UHR mode, which were used for patients with nonsinus rhythm or preoperative evaluation for valvular disease. Among these, 27 underwent CCTA scans performed with ECG-triggered prospective axial acquisition in UHR mode at low tube potentials (70 or 90 kVp). These 27 patients were classified as the PCD_UHR_-low group. Patients were not excluded based on heart rate variability, body weight, or body mass index (BMI). Among these 27 patients, 16 underwent scans at 90 kVp, whereas 11 underwent scans at 70 kVp.

For comparison, 68 patients underwent CCTA at a low tube potential of 70 or 80 kVp using a third-generation dual-source EID-CT scanner (Somatom Force, Siemens Healthineers) at our hospital between October 2023 and May 2024. Two patients with a history of CABG and two patients with retrospective helical scans were excluded, leaving 64 eligible patients. Among these, 27 patients (EID-low) who matched the 27 patients included in the PCD_UHR_-low group were selected using propensity score matching.

**Fig. 1:**
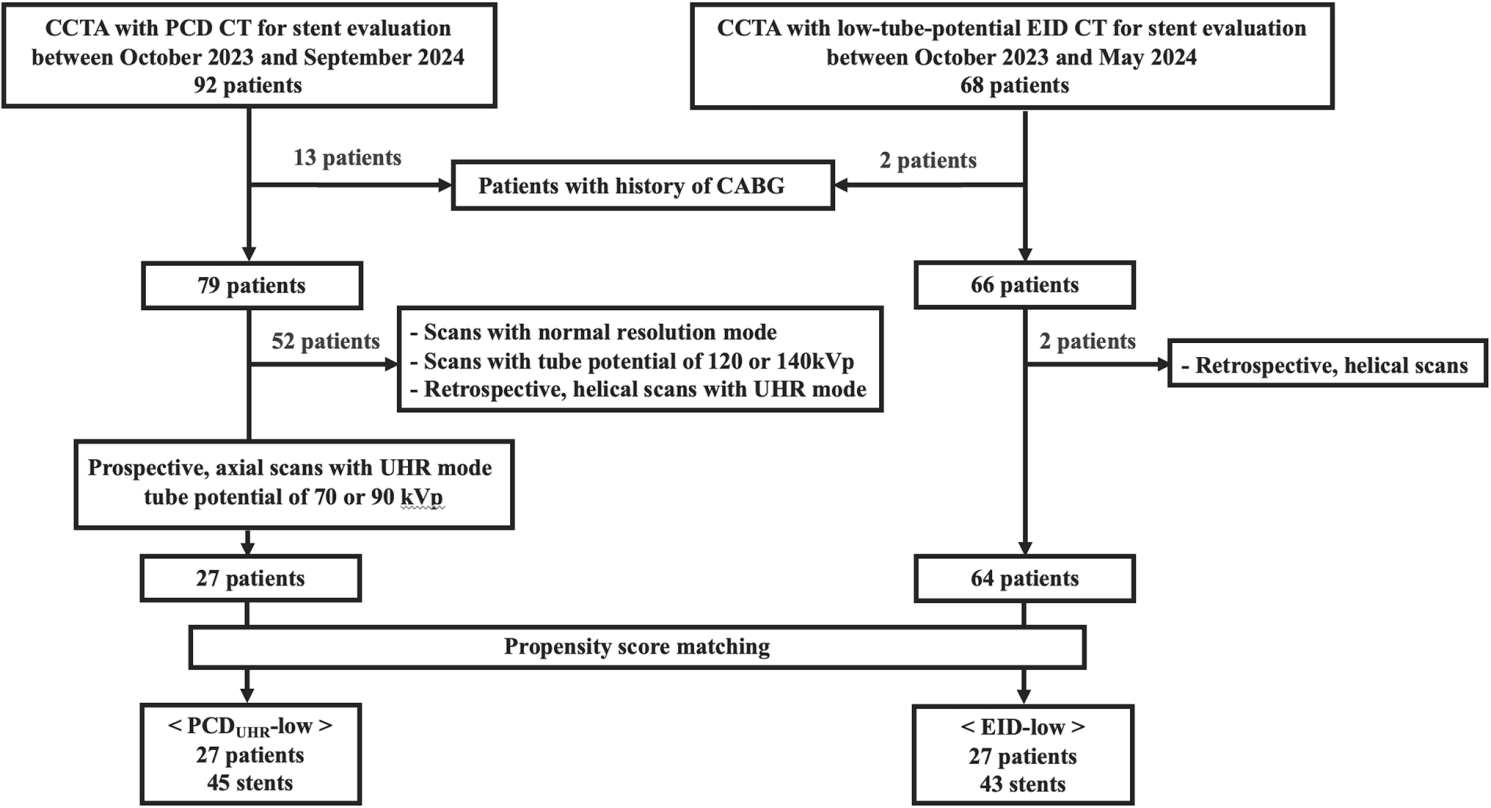
Study flow chart. CCTA, coronary computed tomography angiography; PCD, photon-counting detector; EID, energy-integrating detector; CABG, coronary artery bypass graft; UHR, ultra-high-resolution; PCD_UHR_-low, CCTA scans with ECG-triggered prospective axial acquisition, UHR scan mode, and a low tube potential of 70 or 90 kVp using a PCD-CT scanner; EID-low, CCTA scans with ECG-triggered prospective axial acquisition, and a low tube potential of 70 or 90 kVp using EID-CT

The final study population consisted of 27 patients in the PCD_UHR_-low group, and 27 propensity score-matched patients in the EID-low group. The patient selection process is illustrated in Fig. 1.

In addition, 9 patients who underwent scans with UHR mode at standard tube potentials of 120 or 140 kVp between October 2023 and May 2024 were included in the PCD_UHR_-std group. Among these 9 patients, 2 underwent scans at 140 kVp, whereas 7 underwent scans at 120 kVp. These patients were not included in the propensity score matching but were analyzed separately to evaluate the effects of standard tube potential settings.

### Image acquisition

All scans in PCD_UHR_-low and PCD_UHR_-std were performed using a first-generation dual-source PCD-CT scanner (NAEOTOM Alpha, Siemens Healthineers, Forchheim, Germany), while scans in the EID-low were performed using a third-generation dual-source EID-CT scanner (Somatom Force, Siemens Healthineers). In PCD-CT, CCTA with ECG-triggered prospective axial UHR scan mode was performed with a phase of 65–85% R-R interval by bolus injection of 26 mgI/kg/s of iopamidol (Iopamilon-370, Bayer AG, Leverkusen, Germany) over 12 s, followed by a 20 mL saline flush, with the coronary arteries dilated with a sublingual nitrate (Myocor sprays, Toa Eiyo Co., Ltd., Tokyo, Japan). The acquisition parameters of CCTA in PCD-CT were as follows: collimation of 144 mm × 0.2 mm, gantry rotation time of 0.25 s, and tube voltage of 70 or 90 kVp in PCD_UHR_-low and 120 or 140 kVp in PCD_UHR_-std. Automatic exposure control (CAREkeV, Siemens Healthineers, Forchheim, Germany) was used to determine tube current for each patient. Axial images of CCTA were reconstructed with a slice thickness and increment of 0.2/0.2 mm, quantum iterative reconstruction level of 4, vascular convolution kernel (Bv64), image matrix of 1024 × 1024, and a field-of-view restricted to the heart. The in-plane spatial resolution of PCD-CT in UHR mode was approximately 0.11 mm. Since the PCD-CT system did not allow for the collection of spectral information with UHR mode, polychromatic images called T3D were reconstructed for CCTA with UHR mode.

In EID-CT, ECG-triggered prospective axial CCTA was performed. The contrast medium was injected the same as the protocol of PCD-CT. The acquisition parameters of CCTA in EID-CT were as follows: collimation of 192 mm × 0.6 mm, gantry rotation time of 0.25 s, and tube voltage of 70 or 80 kVp. Automatic exposure control (CAREkVp, Siemens Healthineers, Forchheim, Germany) was used to determine tube current for each patient. Axial images of CCTA were reconstructed with a slice thickness and increment of 0.6/0.6 mm, vascular convolution kernel (Bv49), image matrix of 512 × 512, and a field-of-view restricted to the heart. The in-plane spatial resolution of EID-CT under these reconstruction conditions was approximately 0.24 mm.

Heart rate was controlled before CCTA with intravenous injection of landiolol hydrochloride (Corebeta, Ono Pharmaceutical Co., Ltd., Osaka, Japan), if necessary.

### CCTA image quality analysis

**Fig. 2:**
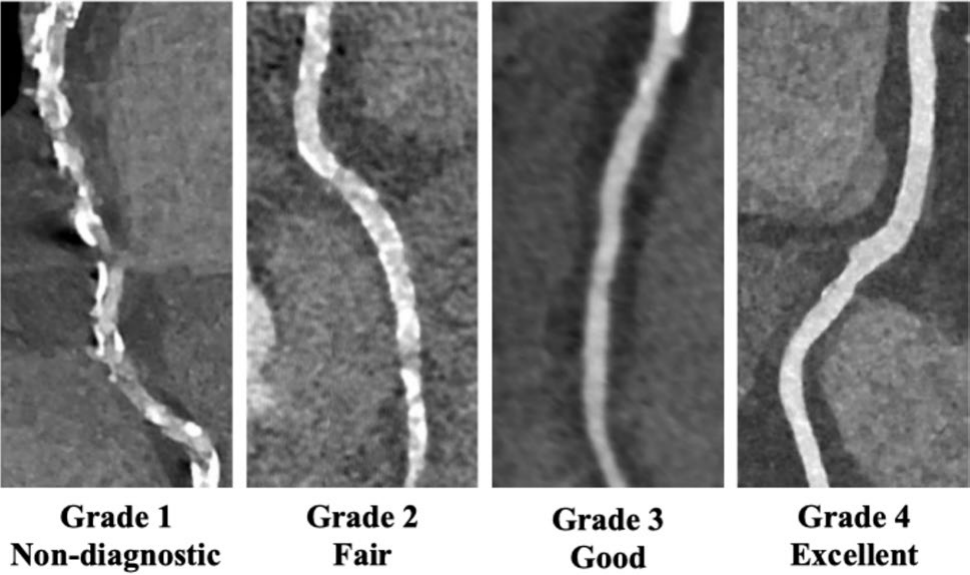
Illustration of image quality scores of CCTA images. The figures show a four-point scale used to assess the image quality of the CCTA images: 1, nondiagnostic; 2, fair; 3, good; and 4, excellent

Although the details of the CCTA image quality assessment are described in the Supplementary Method, briefly, two radiologists, with 4 (S.A.) and 10 (S.N.) years of experience in cardiovascular imaging, performed a consensus visual evaluation of CCTA image quality on a four-point scale (1, nondiagnostic; 2, fair; 3, good; 4, excellent) (Fig. 2), taking into account motion artifacts, sharpness, noise, contrast enhancement, and beam hardening. Objective image quality parameters (signal intensity, image noise, contrast to noise ratio [CNR], and signal to noise ratio [SNR]) were calculated using CT attenuation values from coronary artery lumens and the left ventricular lateral wall.

### Implanted stent image quality analysis

**Fig. 3:**
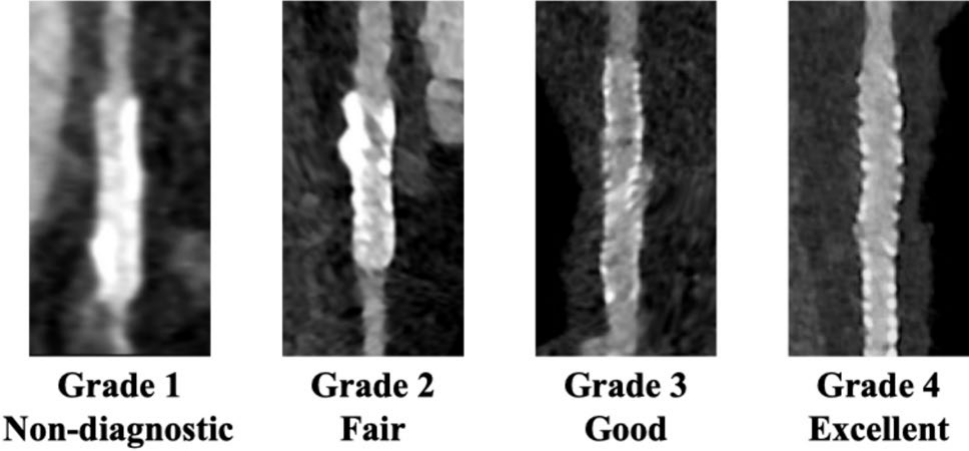
Illustration of image quality scores of in-stent lumens on CCTA images. The figures show a four-point scale used to assess the image quality of the CCTA images: 1, nondiagnostic; 2, fair; 3, good; and 4, excellent

Two radiologists, with 4 (S.A.) and 10 (S.N.) years of experience in cardiovascular imaging, performed a consensus visual assessment of in-stent lumen image quality for each stent using a 4-point Likert scale (1 = nondiagnostic, 2 = fair, 3 = good, 4 = excellent). The evaluation included four specific image quality attributes critical for assessing in-stent morphology and plaque characteristics: motion artifacts, vessel contrast enhancement, sharpness of the stent, and beam hardening artifacts. Each attribute was assessed separately to allow for a more objective comparison between imaging techniques. In addition to these individual parameters, overall image quality was also graded to reflect the general diagnostic acceptability of the in-stent lumen. A score of 1 (“nondiagnostic”) was assigned when severe artifacts prevented the assessment of stent patency. A score of 2 (“fair”) indicated that although artifacts were present, interpretation remained feasible. A score of 3 (“good”) represented minimal interference from artifacts and clear visualization of the stent lumen. A score of 4 (“excellent”) was given when the lumen appeared sharply defined and free of any limiting artifacts (Fig. 3) [19].

To assess the stent-induced blooming, internal and external stent diameters on multiplanar reformatted CT images, focusing on the slice that best represents the stent geometry for each case. When two stents were connected, each stent was measured and analyzed independently. There was no limitation on the maximum number of stents that could be assessed per vessel. The estimated stent-induced blooming was calculated using the following formula [19, 20].

$${\text{Stent - induced blooming }}\left[ \% \right] = \left( {{\text{stent diameter}}_{{{\text{external}}}} \left[ {{\text{mm}}} \right] - {\text{stent diameter}}_{{{\text{internal}}}} \left[ {{\text{mm}}} \right]} \right)/{\text{stent diameter}}_{{{\text{external}}}} \left[ {{\text{mm}}} \right] \times {1}00$$

The effect on in-stent lumen attenuation was also evaluated. Three manually drawn regions of interest (ROIs) were utilized: The first, designated as ROI_in-stent_, was drawn as large as possible to encompass the in-stent lumen while avoiding the vessel walls and stent struts, with a minimum area of 4 mm^2^. Two additional ROIs were placed in the coronary artery lumen—one proximal to the stent (ROI_prox_) and the other distal to the stent (ROI_dist_), both within a maximum distance of 10 mm from the stent edges. From these ROIs, the average Hounsfield unit (HU) values and standard deviation (SD) were measured. Stent struts, vessel walls, and calcified or noncalcified plaques were carefully avoided from the ROIs. With reference to the methods described by Hagar MT, et al. and Boccalini S, et al. [19, 20], ΔHU_in-stent_ were calculated to evaluate the effects of the stent on in-stent lumen attenuation using the three ROIs. ΔHU_in-stent_ quantifies the increase in CT attenuation within the stent lumen. This parameter was derived using the following formulas:

$${\text{ROI}}_{{\text{ex - stent}}} = \left( {{\text{ROI}}_{{{\text{prox}}}} \left[ {{\text{HU}}} \right] + {\text{ROI}}_{{{\text{dist}}}} \left[ {{\text{HU}}} \right]} \right)/{2}$$

$$\Delta {\text{HU}}_{{\text{in - stent}}} = \left( {{\text{ROI}}_{{\text{in - stent}}} \left[ {{\text{HU}}} \right]{-}{\text{ROI}}_{{\text{ex - stent}}} \left[ {{\text{HU}}} \right]} \right)*{1}00/{\text{ROI}}_{{\text{ex - stent}}} \left[ {{\text{HU}}} \right]\left( \% \right)$$

**Fig. 4:**
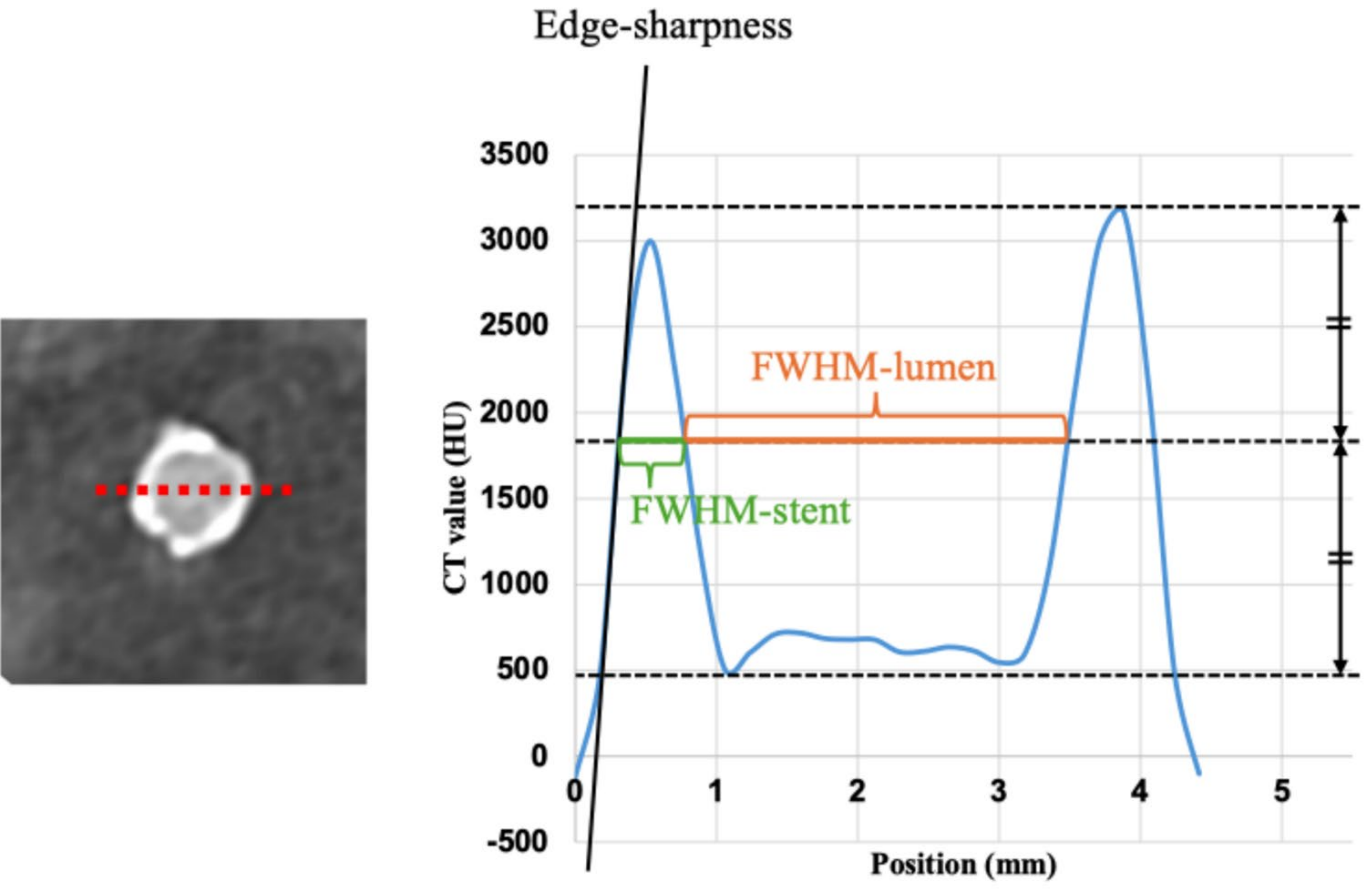
Profile curve shows definition of edge sharpness and full width at half maximum (FWHM). Edge sharpness of stent strut is defined as the maximum slope of the profile curve. FWHMs of lumen (FWHM-lumen) and stent strut (FWHM-stent) were measured

This metric quantitatively captures the stent’s impact on lumen attenuation. Edge sharpness and full width at half maximum (FWHM) of coronary stent were obtained from the vessel profile using the definitions shown in Fig. 4 by a radiologist (S.A.) setting a linear region of interest on a cross-sectional image using the commercially available software (Ziostation2, Ziosoft Inc., Tokyo, Japan) [21].

### Radiation dose estimation

The CT dose index volume (CTDIvol) and dose-length product (DLP) were extracted from the participant protocols.

### Statistical analysis

Descriptive statistics (mean, standard deviation) were used to summarize the data. Student’s t test was used to assess differences in continuous variables between two groups after examining the normality of the data. The chi-squared test was used to assess differences in proportion between categorical variables. Values of p < 0.05 were considered indicative of statistical significance.

Propensity-score matching was performed to select control patients who underwent CCTA with EID-CT and a propensity score was calculated based on age, sex, body height, body weight, and heart rate. Propensity-score matching was performed using the SPSS statistical package (version 23.0, IBM Corp., Armonk, NY, USA). All the other statistical analyses were performed using JMP version 10 software (SAS Institute Inc., Cary, NC, USA).

## Results

### Patient characteristics and radiation dose metrics

Patient characteristics and CCTA imaging parameters are summarized in Table 1. Of the 54 patients included, 38 (70.4%) were male, and the mean age was 70 ± 10 years. The mean body weight was 63.4 ± 14.1 kg, and the mean BMI was 23.8 ± 3.9 kg/m^2^. The mean heart rate during CCTA was 64 ± 8 bpm. There were no significant differences between the PCD_UHR_-low and EID-low groups in terms of patient characteristics, including sex, age, height, weight, and BMI. Regarding radiation exposure, the mean CTDIvol was 9.3 ± 4.4 mGy in the PCD_UHR_-low group and 10.5 ± 5.9 mGy in the EID-low group (*p* = 0.26). Similarly, the mean DLP was 107.1 ± 50.4 mGy·cm and 118.2 ± 74.9 mGy·cm, respectively (*p* = 0.47).

**Table 1:** Patient characteristics, CCTA parameters and image quality of CCTA images

	All participants (*n* = 54)	PCD_UHR_-low (*n* = 27)	EID-low (*n* = 27)	*p* value
Patient characteristics				
Male	38 (70.4)	19 (70.4)	19 (70.4)	N.A
Age [yrs]	70 ± 10	71 ± 2	69 ± 2	0.51
Body height [cm]	162.5 ± 9.8	162.1 ± 8.8	162.7 ± 10.9	0.82
Body weight [kg]	63.4 ± 14.1	62.0 ± 2.8	64.8 ± 2.8	0.48
BMI [kg/m^2^]	23.8 ± 3.9	23.4 ± 0.8	24.2 ± 0.8	0.47
CCTA parameters				
Heart rate [bpm]	64 ± 8	64 ± 2	64 ± 2	0.86
Amount of contrast [ml]	52.5 ± 8.1	54.3 ± 6.8	50.7 ± 9.0	0.11
Flow rate [ml/s]	4.3 ± 0.7	4.4 ± 0.5	4.3 ± 0.8	0.38
CTDIvol [mGy]	9.9 ± 5.2	9.3 ± 4.4	10.5 ± 5.9	0.26
DLP [mGy・cm]	112.7 ± 63.5	107.1 ± 50.4	118.2 ± 74.9	0.47

Data are presented as the mean ± standard deviation or number of patients (%). *p* value indicates comparison of parameters between the groups. *BMI* body mass index, *CCTA* coronary computed tomography angiography, *CTDIvol* computed tomography dose index volume, *DLP* dose-length product

Patient characteristics and CCTA imaging parameters of the PCD_UHR_-std group are summarized in Supplementary Table 1 and 2, accompanied by those of the PCD_UHR_-low group for reference. Regarding radiation dose estimates, the mean CTDIvol and DLP in the PCD_UHR_-std group were 22.5 ± 7.7 mGy and 256.8 ± 83.2 mGy∙cm, respectively, which were significantly higher than those in the PCD_UHR_-low group (*p* < 0.01, both).

### CCTA image quality

Table 2 summarizes the subjective and objective image quality of CCTA. Regarding subjective image quality, among all participants, 36.1% of segments were rated as excellent, 53.3% as good, 9.6% as fair, and 1.0% as nondiagnostic. The PCD_UHR_-low group showed a higher proportion of excellent scores (57.3% vs. 15.3%) and a lower proportion of fair or nondiagnostic scores (3.9% vs. 17.1%) as compared to the EID-low group (*p* < 0.01). Objectively, the PCD_UHR_-low group showed higher image noise (106.7 ± 15.3 HU) than EID-low group, while its signal intensity (782.9 ± 212.9 HU) was comparable to EID-low (*p* = 0.11). The PCD_UHR_-low group had lower CNR and SNR than the EID-low group (both *p* < 0.01). Subjective and objective image quality of CCTA in PCD_UHR_-std are summarized in Supplementary Table 3. Subjective image quality in the PCD_UHR_-std group was comparable to that in the PCD_UHR_-low group, with approximately 95% of segments rated as good or excellent in both groups. SNR and CNR were also comparable between the two groups.

**Table 2:** Image quality of CCTA images

	All participants (*n* = 54)	PCD_UHR_-low (*n* = 27)	EID-low (*n* = 27)	*p* value
Subjective image quality				< 0.01
1. Nondiagnostic	7 (1.0)	4 (1.1)	3 (0.8)	
2. Fair	70 (9.6)	10 (2.8)	60 (16.3)	
3. Good	388 (53.3)	140 (38.8)	248 (67.6)	
4. Excellent	263 (36.1)	207 (57.3)	56 (15.3)	
Objective Image quality				
Signal intensity [HU]	794.8 ± 177.1	782.9 ± 212.9	806.7 ± 135.3	0.11
Image noise [HU]	97.6 ± 17.2	106.7 ± 15.3	88.4 ± 14.0	< 0.01
Contrast-to-noise ratio	8.4 ± 2.2	6.2 ± 2.0	7.3 ± 1.4	0.01
Signal-to-noise ratio	6.7 ± 1.8	7.5 ± 2.2	9.3 ± 1.8	< 0.01

Data are presented as the mean ± standard deviation or number of patients (%). *p* value indicates comparison of parameters between the groups. *HU* Hounsfield unit

### Image quality of implanted stents

Table 3 summarizes the location and caliber of all the implanted stents among the three subgroups. There were no significant differences in stent location (*p* = 0.59) or stent caliber (*p* = 0.85) among the groups. Characteristics of implanted stents in PCD_UHR_-std are summarized in Supplementary Table 4.

**Table 3:** Implanted stent characteristics

	All stents (*n* = 88)	PCD_UHR_-low (*n* = 45)	EID-low (*n* = 43)	*p* value
Stent location				0.59
LMT	4 (4.5)	2 (4.4)	2 (4.7)	
LAD	41 (46.6)	21 (46.7)	20 (46.5)	
LCX	21 (23.9)	13 (28.9)	8 (18.6)	
RCA	22 (25.0)	9 (20.0)	13 (30.2)	
Stent caliber				0.85
≥ 3.5	25 (28.4)	11 (24.5)	14 (32.6)	
3.5 >, ≥ 3.0	27 (30.7)	15 (33.3)	12 (27.9)	
3.0 >, ≥ 2.5	26 (29.5)	14 (31.1)	12 (27.9)	
< 2.5	10 (11.4)	5 (11.1)	5 (11.6)	

Data are presented as the number of patients (%). *p* value indicates comparison of parameters between the groups. *LMT* left main coronary artery, *LAD* left anterior descending artery, *LCX* left circumflex artery, *RCA* right coronary artery

Table 4 summarizes the subjective image quality of implanted stents. No significant differences were observed between the groups in terms of motion artifacts and vessel contrast enhancement (*p* = 0.28 and 0.17, respectively). For motion, only one segment was rated as nondiagnostic in the PCD_UHR_-low group. For contrast, more than 95% of segments were rated as good or excellent in both groups. In contrast, differences were observed in sharpness and beam hardening artifacts. In the PCD_UHR_-low group, 100% of segments were rated as excellent or good for both parameters, whereas in the EID-low group, 60.4% were rated as excellent or good for sharpness, and 90.7% for beam hardening. Subjective image quality of implanted stents in PCD_UHR_-std are summarized in Supplementary Table 5. Subjective image quality of implanted stents was comparable between the PCD_UHR_-low and PCD_UHR_-std groups across all evaluated parameters, including motion, contrast, sharpness, beam hardening, and overall quality.

**Table 4:** Subjective image quality of implanted stents

	All participants (*n* = 88)	PCD_UHR_-low (*n* = 45)	EID-low (*n* = 43)	*p* value
Subjective image quality				
motion				0.28
1. Nondiagnostic	1 (1.1)	1 (2.2)	0 (0)	
2. Fair	8 (9.1)	6 (13.3)	2 (4.7)	
3. Good	5 (5.7)	3 (6.7)	2 (4.7)	
4. Excellent	74 (84.1)	35 (77.8)	39 (90.6)	
Contrast				0.17
1. Nondiagnostic	0 (0)	0 (0)	0 (0)	
2. Fair	2 (2.3)	0 (0)	2 (4.7)	
3. Good	12 (13.6)	5 (11.1)	7 (16.3)	
4. Excellent	74 (84.1)	40 (88.9)	34 (79.0)	
Sharpness				< 0.01
1. Non-diagnostic	3 (3.4)	0 (0)	3 (7.0)	
2. Fair	14 (15.9)	0 (0)	14 (32.6)	
3. Good	17 (19.3)	0 (0)	17 (39.5)	
4. Excellent	54 (61.4)	45 (100)	9 (20.9)	
Beam hardening				< 0.01
1. Nondiagnostic	0 (0)	0 (0)	0 (0)	
2. Fair	4 (4.6)	0 (0)	4 (9.3)	
3. Good	42 (47.7)	18 (40.0)	24 (55.8)	
4. Excellent	42 (47.7)	27 (60.0)	15 (34.9)	

Data are presented as the mean ± standard deviation or number of patients (%). *p* value indicates comparison of parameters between the groups

**Fig. 5:**
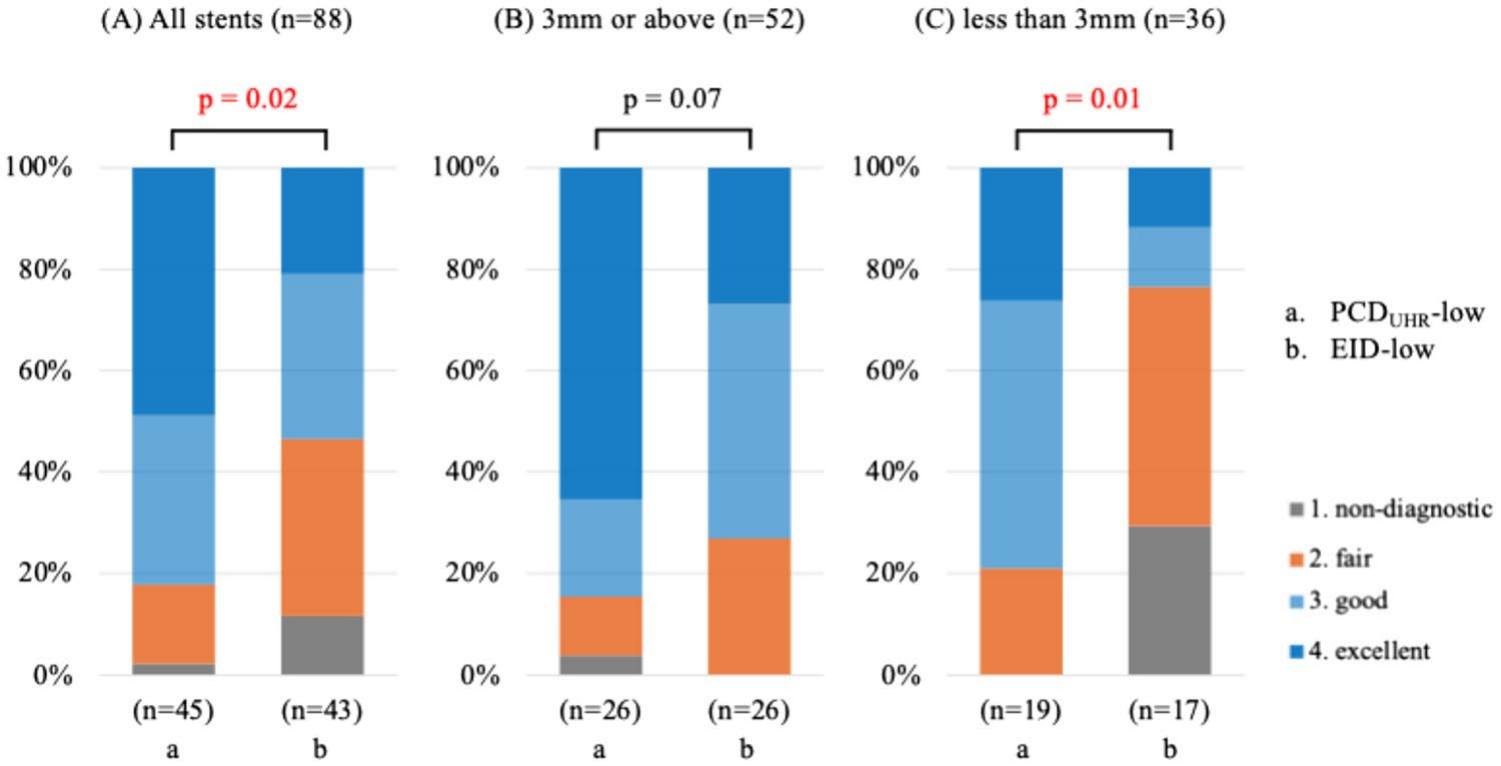
Image quality score of stents. The figure shows distribution of subjective image quality scores based on the Likert scale for (**A**) all stents (*n* = 110), **B** stents with a diameter of ≥ 3 mm (*n* = 63), and **C** stents with a diameter of < 3 mm (*n* = 47). The image quality scores are categorized as 1: non-diagnostic (gray), 2: fair (orange), 3: good (light blue), and 4: excellent (dark blue). Comparisons among groups are shown for PCD_UHR_-low (a), and EID-low (b). There were significant differences for all stents (*p* = 0.02 for PCD_UHR_-low vs. EID-low) and for stents < 3 mm (p = 0.01 for PCD_UHR_-low vs. EID-low), whereas no significant differences were found between groups for stents ≥ 3 mm. PCD_UHR_-low, CCTA scans with ECG-triggered prospective axial acquisition, UHR scan mode, and a low tube potential of 70 or 90 kVp using a PCD-CT scanner; EID-low, CCTA scans with ECG-triggered prospective axial acquisition, and a low tube potential of 70 or 90 kVp using EID-CT

Figure 5 illustrates the distribution of subjective overall image quality scores of implanted stents. For all stents (*n* = 88), the proportions of segments rated as “excellent” (score 4), “good” (score 3), “fair” (score 2), and “nondiagnostic” (score 1) were 35.2% (*n* = 31), 33.0% (*n* = 29), 25.0% (*n* = 22), and 6.8% (*n* = 6), respectively. When stratified by group, in the PCD_UHR_-low group (*n* = 45), the proportions were 48.9% (score 4, *n* = 22), 33.3% (score 3, *n* = 15), 15.6% (score 2, *n* = 7), and 2.2% (score 1, *n* = 1). In the EID-low group (*n* = 43), the proportions were 20.9% (score 4, *n* = 9), 32.6% (score 3, *n* = 14), 34.9% (score 2, *n* = 15), and 11.6% (score 1, *n* = 5). The PCD_UHR_-low group demonstrated a significantly better image quality distribution compared to the EID-low group (*p* = 0.02). When stents were stratified by diameter, the differences in image quality distribution became more apparent. For stents with a diameter of ≥ 3 mm (*n* = 52), no significant differences were observed between the PCD _UHR_-low and EID-low groups (*p* = 0.07). However, for stents with a diameter of < 3 mm (*n* = 36), the PCD_UHR_-low group demonstrated a significantly better image quality distribution compared to the EID-low group (p = 0.01).

The objective image quality metrics specific to implanted stents are detailed in Table 5, Supplementary Table 6 and Supplementary Table 7. For all stents, the mean stent-induced blooming was 37.4 ± 14.6%. ΔHU_in-stent_ was 22.1 ± 22.9%. The PCD_UHR_-low group demonstrated significantly lower stent-induced blooming (28.1 ± 9.8%) as compared to the EID-low group (47.1 ± 12.2%, *p* < 0.01), as well as a lower ΔHU_in-stent_ (15.1 ± 13.4%) as compared to the EID-low group (29.4 ± 28.1%, *p* < 0.01). The objective image quality metrics in PCD_UHR_-std are summarized in Supplementary Table 8. In the PCD_UHR_-std group, ΔHU_in-stent_ was significantly lower than in the PCD_UHR_-low group, while other objective image quality metrics were comparable between the two groups.

**Table 5:** Objective image quality of implanted stent (all)

	All stents (*n* = 88)	PCD_UHR_-low (*n* = 45)	EID-low (*n* = 43)	*p* value
Stent-induced blooming [%]	37.4 ± 14.6	28.1 ± 9.8	47.1 ± 12.2	< 0.01
ΔHU_in_stent_ [%]	22.1 ± 22.9	15.1 ± 13.4	29.4 ± 28.1	< 0.01
Edge sharpness [HU/mm]	4792.0 ± 3068.3	7178.2 ± 2550.5	2294.8 ± 448.8	< 0.01
FWHM-stent [mm]	0.61 ± 0.17	0.50 ± 0.11	0.71 ± 0.15	< 0.01
FWHM-lumen [mm]	2.19 ± 0.62	2.36 ± 0.56	2.02 ± 0.64	< 0.01

Data are presented as the mean ± standard deviation. *p* value indicates comparison of parameters between the groups. *FWHM* full width at half maximum

**Fig. 6:**
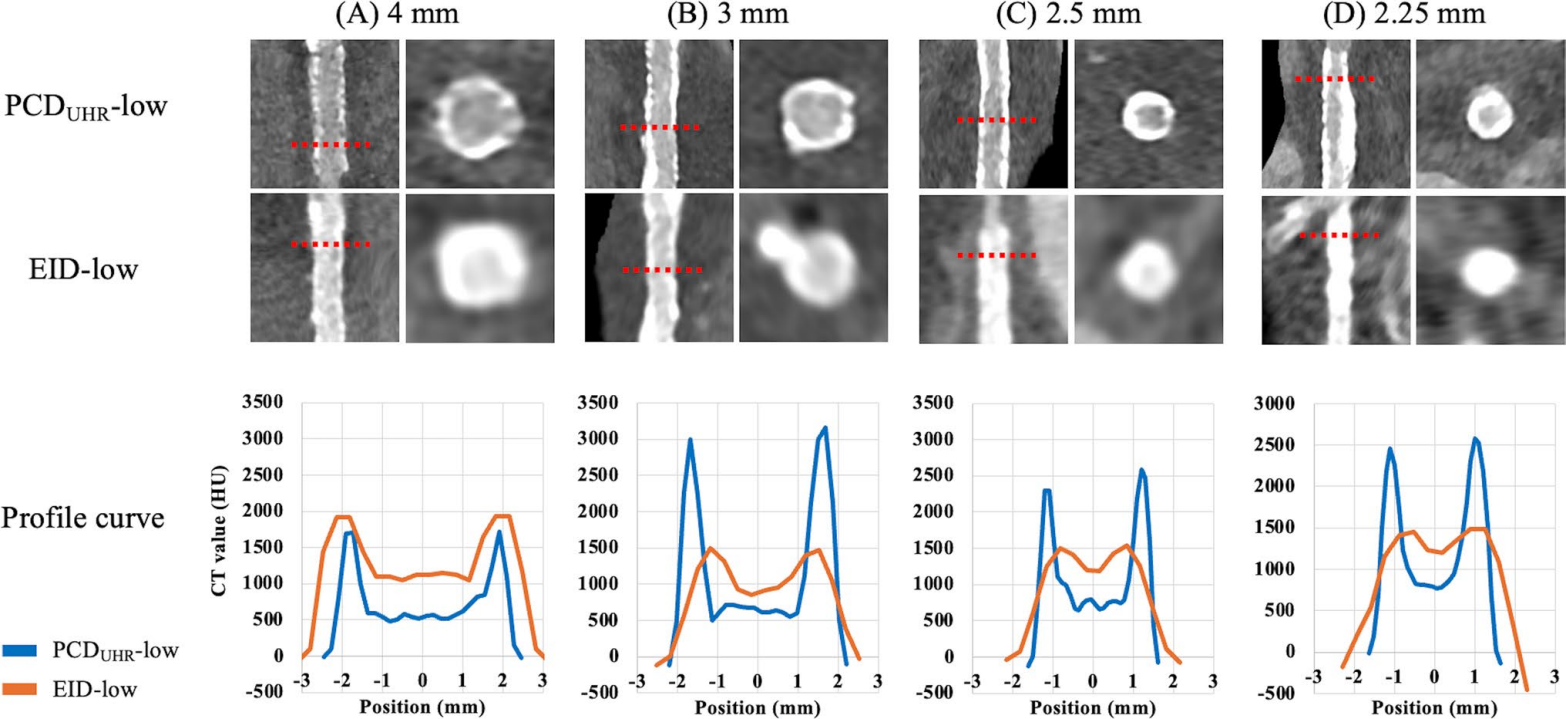
Representative cases of stents with different diameters. The figures show representative images and attenuation profile curves for different stent diameters (**A**: 4 mm, **B**: 3 mm, **C**: 2.5 mm, **D**: 2.25 mm) between PCD_UHR_-low and EID-low. The top row displays cross-sectional and longitudinal images of the stents between the groups. The middle row shows attenuation profile curves, indicating thinner stent struts (narrower FWHM-stent) and larger lumen diameters (wider FWHM-lumen) in the PCD_UHR_-low group as compared to the EID-low groups. PCD_UHR_-low, CCTA scans with ECG-triggered prospective axial acquisition, UHR scan mode, and a low tube potential of 70 or 90 kVp using a PCD-CT scanner; EID-low, CCTA scans with ECG-triggered prospective axial acquisition, and a low tube potential of 70 or 90 kVp using EID-CT; FWHM, full width at half maximum

Figure 6 illustrates representative cases of group-wise comparisons stratified by stent diameter. The upper row demonstrates the appearance of stents in the two groups, highlighting the visual differences in stent depiction. The lower row shows attenuation profile curves, which reveal that stents in the PCD_UHR_-low group are depicted with thinner stent struts (as indicated by smaller FWHM-stent values) and larger lumen diameters (as indicated by larger FWHM-lumen values) as compared to the EID-low group.

## Discussion

For the primary analysis of implanted stents, the PCD_UHR_-low group had significantly better overall subjective image quality with superior ratings for sharpness and beam hardening artifacts than the EID-low group; this benefit was particularly pronounced in stents with a diameter of less than 3 mm. Objectively, the PCD_UHR_-low protocol resulted in significantly less stent-induced blooming and superior edge sharpness as compared to the EID-low protocol. When compared to the PCD_UHR_-std group, the PCD_UHR_-low protocol provided comparable image quality but at a significantly lower radiation dose.

EID-CT has demonstrated efficacy in evaluating coronary artery stents, particularly with advancements in multidetector row technology [22]. Studies using 320-row EID-CT have shown acceptable diagnostic accuracy for in-stent restenosis, suggesting a potential non-invasive alternative to invasive coronary angiography [9]. However, EID-CT has limitations in stent evaluation due to artifacts and limited spatial resolution, especially in smaller stents or cases with severe blooming artifacts from metal stent struts, which can reduce diagnostic accuracy [11]. Previous studies have reported that 8–28% of stent lumens cannot be assessed due to stent-induced artifacts [5, 9, 23–25], primarily caused by partial volume averaging and beam hardening. Consequently, the effectiveness of CCTA using EID-CT in patients with previous stent implantation, particularly those with smaller diameter stents, remains controversial due to the rate of unassessable stents.

To date, no consensus has been reached regarding the optimal tube voltage for coronary stent imaging with EID-CT, although 120 kVp is typically used; higher tube voltages, such as 140 kVp, have been employed in some cases due to their ability to penetrate dense stent struts and reduce blooming artifacts [10], but this approach can increase radiation exposure, which is a significant concern for patients undergoing frequent imaging [4]. However, some reports have shown that lowering the tube voltage can improve the CNR, thereby enhancing stent lumen visibility; studies by Eisentopf et al. [24] and Lee et al. [25] suggested that lower tube voltages (80 to 100 kVp) can provide comparable image quality to that produced by higher tube voltages for stent assessment while reducing radiation doses, offering a viable alternative for routine clinical practice.

PCD-CT have represented a significant advancement in cardiac CT, offering substantial improvements in image quality and noise reduction. This technology, which directly converts X-ray photons into electronic signals, employs smaller detector elements that enable UHR imaging and achieves superior SNR by effectively eliminating electronic noise [12]. Studies such as Geering et al. [26] and von Spiczak et al. [16] have demonstrated that UHR mode offered superior spatial resolution, potentially enabling clearer visualization of stent struts and more accurate assessment of in-stent restenosis compared to EID-CT. These benefits are attributed not only to the increased spatial resolution provided by smaller detector elements, but also to the use of dedicated sharp convolution kernels, which may help reduce blooming artifacts and enhance stent lumen delineation. In the present study, the use of PCD-CT, as compared to EID-CT, also demonstrated a significant reduction in stent-induced artifacts.

In this study, low-tube-potential UHR CCTA using PCD-CT (PCD_UHR_-low) demonstrated clear superiority over low-tube-potential EID-CT (EID-low) in coronary stent evaluation. Notably, this superiority was evident in overall image quality, including subjective assessments and several objective measures, despite the comparable radiation dose between the two protocols. Importantly, EID-low has been validated in recent studies as a clinically feasible approach for stent evaluation, owing to advancements in image reconstruction techniques such as deep learning and sharp kernel methods [27, 28]. Against this validated reference, PCD_UHR_-low provided further improvements in image quality. Specifically, it effectively reduced blooming artifacts and improved the visualization of the stent lumen, likely owing to the intrinsic advantages of PCD technology, such as smaller detector elements and the elimination of electronic noise. The PCD-CT group exhibited higher image noise (SD) and lower SNR and CNR compared with the EID-CT group; however, these objective differences can be largely attributed to the employment of a sharper reconstruction kernel (Bv64 vs. Bv49), which enhances spatial resolution at the cost of increased noise. Importantly, overall image quality must integrate both quantitative and qualitative aspects. In our combined evaluation, PCD-CT’s superior spatial resolution and visually sharp stent delineation resulted in higher subjective scores, compensating for its increased noise. This finding indicates the necessity of balancing objective noise measures against subjective image‐quality characteristics when optimizing UHR CCTA protocols. By providing higher-quality images with fewer artifacts while maintaining a low radiation dose, low-tube-potential UHR CCTA using PCD-CT may enable more accurate and reliable detection of in-stent restenosis, potentially contributing to more appropriate and efficient selection of patients for invasive coronary angiography. On the other hand, low-tube-potential UHR CCTA provided comparable image quality for stent evaluation while achieving a significantly lower radiation dose. In our cohort, the mean DLP of the low-tube-potential UHR CCTA was 107.1 ± 50.4 mGy∙cm, significantly lower than that of the standard UHR protocol (256.8 ± 83.2 mGy∙cm). This reduction is also notable when compared with previous reports of UHR CCTA using standard tube potentials, with previously reported DLP values ranging from approximately 240 to 936 mGy∙cm [29–32].

This study has several limitations. First, it was a single-center retrospective investigation, which may limit the generalizability of the findings. Second, the imaging modes were not compared within the same patients, potentially introducing selection bias and limiting the robustness of direct comparisons. Third, different reconstruction kernels were used for PCD-CT and EID-CT, which might have influenced stent evaluation, including blooming artifacts and lumen visibility. In this study, we prioritized selecting the reconstruction kernels that were considered most appropriate for stent evaluation with each scanner [13, 24, 33]. Forth, different stent materials may result in varying levels of x-ray attenuation and thus could have influenced image quality. However, because we lacked complete data on the stent materials used in some patients, we neither classified stents according to their propensity to produce artifacts nor performed statistical analyses comparing image quality by stent material. Moreover, even if we restricted our analysis to those stents with known materials, the wide variety of stent types would necessitate dividing them into multiple subgroups, which would likely result in insufficient statistical power. The fifth limitation is the lack of standardization in tube voltage selection between the two CT systems. This reflects the retrospective nature of the study, where tube voltage was determined according to routine clinical protocols, and available voltage options differed between the systems. Such differences may have influenced image quality metrics, particularly noise and contrast. Sixth, although our preliminary retrospective analysis suggests that stent visualization in PCD_UHR_-low may be comparable to that at PCD_UHR_-std, the small sample size of the PCD_UHR_-std group and the lack of matched baseline characteristics between the two groups limits the strength and generalizability of these observations. Future work should therefore include prospective imaging of the same patients across a spectrum of tube voltages under standardized conditions or, alternatively, validation using a phantom model. Such studies will be critical to establish the optimal tube voltage in UHR CCTA. Finally, the absence of a reference standard such as invasive angiography leaves uncertainty regarding the true accuracy of the imaging findings and warrants further validation by standardized reference assessments.

## Conclusion

This study demonstrated that the low-tube-potential UHR mode in PCD-CT offers a favorable balance between dose reduction and image quality improvement in coronary stent imaging. Compared with low-tube-potential imaging with EID-CT, this approach achieved similar radiation doses while yielding superior subjective and objective image quality, including fewer stent-related artifacts. These findings suggested the importance of optimizing tube voltage in UHR mode to achieve safer, more accurate coronary stent assessments.

## Supplementary Information

Below is the link to the electronic supplementary material.

Supplementary file1 (DOCX 30 KB)
