# Supplementary material for: Low-tube-potential ultra-high-resolution coronary CTA with photon-counting detector CT for stent evaluation: a comparative feasibility study
Source: Jpn J Radiol. 2025 Aug 9;43(12):1981–92. doi: 10.1007/s11604-025-01846-x (PMC12647213; doi:10.1007/s11604-025-01846-x)
Supplement: Supplementary file 1 — Supplementary file1 (DOCX 30 KB) [file 11604_2025_1846_MOESM1_ESM.docx]

**Supplementary method**

Two radiologists, with 4 (S.A.) and 10 (S.N.) years of experience in cardiovascular imaging, conducted a consensus reading to visually assess the image quality of the CCTA data. The observers used the following four-point scale for the visual assessment, considering factors such as motion artifacts, sharpness, noise, contrast enhancement, and beam hardening, which are crucial for analyzing the morphology of coronary arteries and plaque: 1, nondiagnostic (the image quality is so poor that assessing the morphology of coronary arteries and plaque is impossible); 2, fair (the image quality poses some challenges, but morphology assessment is still feasible); 3, good (the image quality presents minor imperfections, yet allows for morphology assessment without major difficulties); and 4, excellent (the image quality makes assessment straightforward and easy) (Figure 2) [1,2]. In this visual assessment, the observers evaluated each segment of the coronary arteries based on the 18-segment model of the Society of Cardiovascular Computed Tomography [3]. Signal intensity, image noise, contrast-to-noise ratio (CNR) and the signal-to-noise ratio (SNR) were quantified as objective image quality parameters. All measurements were performed (by S.A.) on reformatted axial images with a slice thickness of 0.2mm in image of PCD-CT or 0.6mm in image of EID-CT. Signal intensity was derived from the mean CT attenuation values (Hounsfield units; HU) averaged from 2 circular regions of interest (size 3-4 mm2) in the proximal segments of the left and right coronary artery lumen. Image noise was defined as the averaged standard deviations of the CT attenuation values within these 2 regions of interest. We calculated the difference between the mean CT attenuation values of the proximal coronary arteries and the mean CT attenuation value of the left ventricular lateral wall and defined the CNR as the difference divided by image noise. The SNR was calculated as mean CT attenuation values of the left and right coronary arteries divided by the image noise [4].

References

1. Stocker TJ, Leipsic J, Chen MY, Achenbach S, Knuuti J, Newby D, Hausleiter J. Influence of Heart Rate on Image Quality and Radiation Dose Exposure in Coronary CT Angiography. Radiology. 2021;300, 701–703.

2. Araki S, Nakamura S, Takafuji M, Ichikawa Y, Sakuma H, Kitagawa K. Ultra-low-dose coronary computed tomography angiography using photon-counting detector computed tomography. Eur Heart J Imaging Methods Pract. 2024;2(3):qyae125.

3. Leipsic J, Abbara S, Achenbach S, Cury R, Earls JP, Mancini GJ et al. SCCT guidelines for the interpretation and reporting of coronary CT angiography: a report of the Society of Cardiovascular Computed Tomography Guidelines Committee. J Cardiovasc Comput Tomogr 2014;8:342–58.

4. Hausleiter J, Martinoff S, Hadamitzky M, et al. (2010) Image quality and radiation exposure with a low tube voltage protocol for coronary CT angiography results of the PROTECTION II Trial. J Am Coll Cardiol Img. 3(11):1113-1123.

**Supplementary Table 1. Patient characteristics and CCTA parameters of CCTA images**

|  | PCD_UHR_-low  (n=27) | PCD_UHR_-std  (n=9) | p-value |
| --- | --- | --- | --- |
| Patient characteristics |  |  |  |
| Male | 19 (70.4) | 7 (77.8) | 0.67 |
| Age [yrs] | 71 ±2 | 67 ± 3 | 0.37 |
| Body height [cm] | 162.1 ± 8.8 | 165.0 ± 8.7 | 0.65 |
| Body weight [kg] | 62.0 ± 2.8 | 72.4 ± 4.9 | 0.13 |
| BMI [kg/m^2^] | 23.4 ± 0.8 | 26.5 ± 1.4 | 0.22 |
| CCTA parameters |  |  |  |
| Heart rate [bpm] | 64 ± 2 | 62 ± 3 | 0.34 |
| Amount of contrast [ml] | 54.3 ± 6.8 | 55.7 ± 4.9 | 0.60 |
| Flow rate [ml/s] | 4.4 ± 0.5 | 4.6 ± 0.4 | 0.38 |
| CTDIvol [mGy] | 9.3 ± 4.4 | 22.5 ± 7.7 | < 0.01 |
| DLP [mGy・cm] | 107.1 ± 50.4 | 256.8 ± 83.2 | < 0.01 |

Data are presented as the mean ± standard deviation or number of patients (%). p-value indicates comparison of parameters between the groups. BMI, body mass index; CCTA, coronary computed tomography angiography; CTDIvol, computed tomography dose index volume; DLP, dose-length product.

**Supplementary Table 2. Patient characteristics and CCTA parameters of CCTA images in PCD_UHR_-std group, stratified by tube potential**

|  | 120kVp  (n=7) | 140kVp  (n=2) |
| --- | --- | --- |
| Patient characteristics |  |  |
| Male | 5 (71.4) | 2 (100) |
| Age [yrs] | 67 ± 15 | 65.5 ± 12 |
| Body height [cm] | 163.0 ± 7.7 | 172.2 ± 11.0 |
| Body weight [kg] | 75.5 ± 19.6 | 61.5 ± 5.7 |
| BMI [kg/m^2^] | 28.2 ± 5.9 | 20.7 ± 0.7 |
| CCTA parameters |  |  |
| Heart rate [bpm] | 62 ± 11 | 62 ± 13 |
| Amount of contrast [ml] | 56.6 ± 4.6 | 52.5 ± 6.4 |
| Flow rate [ml/s] | 4.7 ± 0.4 | 4.4 ± 0.5 |
| CTDIvol [mGy] | 22.3 ± 8.6 | 19.7 ± 3.7 |
| DLP [mGy・cm] | 264.4 ± 94.3 | 230.0 ± 14.1 |

Data are presented as the mean ± standard deviation or number of patients (%). p-value indicates comparison of parameters between the groups. BMI, body mass index; CCTA, coronary computed tomography angiography; CTDIvol, computed tomography dose index volume; DLP, dose-length product.

**Supplementary Table 3. Image quality of CCTA images**

|  | PCD_UHR_-low (n=27) | PCD_UHR_-std  (n=9) | p-value |
| --- | --- | --- | --- |
| Subjective Image quality |  |  | < 0.01 |
| 1. non-diagnostic | 4 (1.1) | 1 (0.8) |  |
| 2. fair | 10 (2.8) | 8 (6.2) |  |
| 3. good | 140 (38.8) | 31 (23.8) |  |
| 4. excellent | 207 (57.3) | 90 (69.2) |  |
| Objective Image quality |  |  |  |
| Signal intensity [HU] | 782.9 ± 212.9 | 557.4 ± 77.8 | < 0.01 |
| Image noise [HU] | 106.7 ± 15.3 | 70.5 ± 12.7 | < 0.01 |
| Contrast-to-noise ratio | 6.2 ± 2.0 | 6.4 ± 1.0 | 0.41 |
| Signal-to-noise ratio | 7.5 ± 2.2 | 8.0 ± 1.2 | 0.33 |

Data are presented as the mean ± standard deviation or number of patients (%). p-value indicates comparison of parameters between the groups. HU, Hounsfield unit.

**Supplementary Table 4. Implanted stent characteristics**

|  | PCD_UHR_-low  (n=45) | PCD_UHR_-std  (n=22) | p-value |
| --- | --- | --- | --- |
| Stent location |  |  | 0.10 |
| LMT | 2 (4.4) | 2 (9.1) |  |
| LAD | 21 (46.7) | 8 (36.4) |  |
| LCX | 13 (28.9) | 2 (9.1) |  |
| RCA | 9 (20.0) | 10 (45.4) |  |
| Stent caliber |  |  | 0.06 |
| ≥3.5 | 11 (24.5) | 8 (36.4) |  |
| 3.5>, ≥3.0 | 15 (33.3) | 3 (13.6) |  |
| 3.0>, ≥2.5 | 14 (31.1) | 4 (18.2) |  |
| <2.5 | 5 (11.1) | 7 (31.8) |  |

Data are presented as the number of patients (%). p-value indicates comparison of parameters between the groups. LMT, left main coronary artery; LAD, left anterior descending artery; LCX, left circumflex artery; RCA, right coronary artery

**Supplementary Table 5. Subjective image quality of implanted stents**

|  | PCD_UHR_-low (n=45) | PCD_UHR_-std  (n=22) | p-value |
| --- | --- | --- | --- |
| Subjective Image quality |  |  |  |
| motion |  |  | 0.42 |
| 1. non-diagnostic | 1 (2.2) | 0 (0) |  |
| 2. fair | 6 (13.3) | 1 (4.6) |  |
| 3. good | 3 (6.7) | 3 (13.6) |  |
| 4. excellent | 35 (77.8) | 18 (81.8) |  |
| contrast |  |  | 0.43 |
| 1. non-diagnostic | 0 (0) | 0 (0) |  |
| 2. fair | 0 (0) | 0 (0) |  |
| 3. good | 5 (11.1) | 4 (16.3) |  |
| 4. excellent | 40 (88.9) | 18 (79.0) |  |
| sharpness |  |  | N.A. |
| 1. non-diagnostic | 0 (0) | 0 (0) |  |
| 2. fair | 0 (0) | 0 (0) |  |
| 3. good | 0 (0) | 0 (0) |  |
| 4. excellent | 45 (100) | 22 (100) |  |
| beam hardening |  |  | 0.94 |
| 1. non-diagnostic | 0 (0) | 0 (0) |  |
| 2. fair | 0 (0) | 0 (0) |  |
| 3. good | 18 (40.0) | 9 (40.9) |  |
| 4. excellent | 27 (60.0) | 13 (59.1) |  |
| overall |  |  |  |
| 1. non-diagnostic | 1 (2.2) | 0 (0) | 0.18 |
| 2. fair | 7 (15.6) | 1 (4.6) |  |
| 3. good | 15 (33.3) | 13 (59.1) |  |
| 4. excellent | 22 (48.9) | 8 (36.3) |  |

Data are presented as the mean ± standard deviation or number of patients (%). p-value indicates comparison of parameters between the groups.

**Supplementary Table 6. Image quality of implanted stent (3mm or above)**

|  | All stents  (n=52) | PCD_UHR_-low  (n=26) | EID-low  (n=26) | p-value |
| --- | --- | --- | --- | --- |
| Stent-induced blooming [%] | 33.0 ± 11.7 | 24.5 ± 7.4 | 41.5 ± 8.7 | < 0.01 |
| ΔHU_in_stent_ [%] | 13.1 ± 11.7 | 7.5 ± 9.0 | 18.6 ± 11.6 | < 0.01 |
| Edge sharpness [HU/mm] | 4548.0 ± 2879.7 | 6847.4 ± 2392.7 | 2248.5 ± 440.8 | < 0.01 |
| FWHM-stent [mm] | 0.62 ± 0.18 | 0.52 ± 0.12 | 0.73 ± 0.16 | < 0.01 |
| FWHM-lumen [mm] | 2.50 ± 0.55 | 2.69 ± 0.48 | 2.32 ± 0.56 | < 0.01 |

Data are presented as the mean ± standard deviation. p-value indicates comparison of parameters between the groups. FWHM, full width at half maximum.

**Supplementary Table 7. Image quality of implanted stent (less than 3mm)**

|  | All stents  (n=36) | PCD_UHR_-low  (n=19) | EID-low  (n=17) | p-value |
| --- | --- | --- | --- | --- |
| Stent-induced blooming [%] | 43.6 ± 16.1 | 32.9 ± 10.8 | 55.6 ± 12.1 | < 0.01 |
| ΔHU_in_stent_ [%] | 35.2 ± 28.4 | 25.5 ± 11.4 | 46.0 ± 37.2 | 0.15 |
| Edge sharpness [HU/mm] | 5144.4 ± 3331.8 | 7630.7 ± 2752.2 | 2365.6 ± 465.1 | < 0.01 |
| FWHM-stent [mm] | 0.58 ± 0.15 | 0.49 ± 0.09 | 0.69 ± 0.13 | < 0.01 |
| FWHM-lumen [mm] | 1.75 ± 0.40 | 1.90 ± 0.28 | 1.57 ± 0.45 | 0.01 |

Data are presented as the mean ± standard deviation. p-value indicates comparison of parameters between the groups. FWHM, full width at half maximum.

**Supplementary Table 8. Objective image quality of implanted stent**

|  | PCD_UHR_-low (n=45) | PCD_UHR_-std  (n=22) | p-value |
| --- | --- | --- | --- |
| Stent-induced blooming [%] | 28.1 ± 9.8 | 30.7 ± 9.7 | 0.38 |
| ΔHU_in_stent_ [%] | 15.1 ± 13.4 | 6.9 ± 17.2 | < 0.01 |
| Edge sharpness [HU/mm] | 7178.2 ± 2550.5 | 8247.4 ± 3117.1 | 0.14 |
| FWHM-stent [mm] | 0.50 ± 0.11 | 0.56 ± 0.10 | 0.14 |
| FWHM-lumen [mm] | 2.36 ± 0.56 | 2.42 ± 0.77 | 0.67 |

Data are presented as the mean ± standard deviation. p-value indicates comparison of parameters between the groups. FWHM, full width at half maximum.
